# Bayesian Decision-Making Shapes Phenotypic Landscapes from Differentiation to Cancer

**DOI:** 10.3390/e28030312

**Published:** 2026-03-10

**Authors:** Arnab Barua, Haralampos Hatzikirou

**Affiliations:** 1 Center for Information Services and High Performance Computing, Technische Univesität Dresden, Nöthnitzer Straße 46, 01062 Dresden, Germany; 2 Mathematics Department, Khalifa University, Abu Dhabi P.O. Box 127788, United Arab Emirates

**Keywords:** cell decision-making, Bayesian learning, dynamical systems, Fokker-Planck equation, phenotypic dynamics, cancer, differentiation

## Abstract

Cells adapt their phenotypes in noisy microenvironments while maintaining robust decision-making. We develop a coarse-grained theoretical framework in which cellular phenotypic adaptation is described as Bayesian decision-making coupled to replication and diffusion. This leads to an effective Fokker-Planck equation with an emergent fitness landscape governing phenotypic dynamics. We identify distinct phenotypic regimes homeostatic fixation, bistable decision-making, critical switching, and runaway explosion and propose a biological interpretation in which homeostatic and bistable landscapes correspond to healthy differentiated cell states, whereas explosive landscapes capture stem-like or cancer-like behavior. In the Gaussian setting, the correlation between intrinsic and extrinsic states directly encodes mutual information and acts as a bifurcation parameter: high correlation produces shallow or explosive landscapes associated with phenotypic plasticity, while reduced correlation stabilizes differentiated fates by deepening potential wells. We further show that proliferation reshapes these landscapes in a nontrivial manner. Proliferation conditionally stabilizes local homeostasis without altering global confinement, or cooperates with biased environmental sensing to eliminate homeostasis/bistability and drive cancer-like phenotypic explosion even at high phenotypic fidelity. Finally, we show that negative intrinsic–extrinsic correlations suppress explosive dynamics but also reduce bistable plasticity, suggesting a robustness–plasticity trade-off. Together, our results suggest that development, tissue homeostasis, and carcinogenesis can be understood as information-driven deformations of a Bayesian phenotypic fitness landscape.

## 1. Introduction

Decision-making consists of identifying objectives and choosing actions that optimize outcomes among available alternatives [1]. In a similar spirit, cells can be regarded as decision-making agents that adapt their behavior through phenotypic evolution in response to local microenvironmental cues, while operating under fixed intrinsic regulatory wiring. Cells sense information from extrinsic variables such as ligands, nutrients, cell density, or mechanical stress, which is encoded into internal states via biophysical and biochemical processes. This information is subsequently decoded into phenotypic responses, creating a feedback loop in which cellular decisions reshape the microenvironment [2,3,4,5,6].

A defining characteristic of cellular decision-making is its stochastic nature. Cells operate in noisy environments and experience fluctuations arising from low copy numbers of molecular species, environmental variability, and intrinsic stochasticity in biochemical reactions [7]. Noise is not purely detrimental; it can play a constructive role in probabilistic decision-making, generate phenotypic heterogeneity, and support bet-hedging strategies [8,9,10]. The filtering, amplification, and utilization of noise in signaling pathways have been extensively discussed in previous theoretical and experimental studies [11,12]. At the same time, phenotypic decisions are regulated through the coordinated interaction of gene regulatory networks [13,14], signaling pathways [15], and metabolic states that integrate external cues with internal dynamics [16]. Extrinsic signals sensed by receptors are transduced into intracellular signaling dynamics, which modulate transcriptional and epigenetic programs to stabilize specific phenotypic states or enable transitions between them [17,18]. Feedback loops, multistability, and threshold mechanisms within these networks allow cells to commit to discrete fates while retaining flexibility in changing environments [19,20]. Although phenotypic transitions have been traditionally analyzed in the context of dynamical systems and epigenetic lanscape (e.g., [21,22,23]), the aspect of how microenvironmental information is processed by cells leading to these phenotypic decisions has been missing.

Despite significant progress in identifying molecular mechanisms, a complete understanding of phenotypic control at a coarse-grained level remains elusive. In many biological systems, intracellular regulatory interactions are only partially characterized, and the relevant variables are high dimensional and noisy; often it is considered as a “black box” [24,25]. This raises several fundamental questions: How can cellular phenotypic control be characterized in a reduced, probabilistic framework? How do reliable decisions emerge from stochastic intracellular regulation and noisy extracellular signals? How do cells achieve robust phenotypic outcomes despite environmental uncertainty? And how does sensing accuracy or its imperfection shape phenotypic precision, stability, and the emergence of pathological states such as malignancy? To answer the latter, we postulate our main biological assumption that differentiated cells can transition back to an undifferentiated, stem-like cell state which can explain carcinogenesis [26]. This reversion was first discovered by Takahashi and Yamanaka [27] where any differentiated cell can be reprogrammed to a pluripotent state by expressing appropriate transcription factors (the Nobel Prize in Medicine 2012).

To address these questions, we adopt *Bayesian learning* as a candidate organizing principle for cellular phenotypic adaptation. Although people used the idea of Bayesian inference at the level of cellular decision-making and developmental patterning particularly in the context of morphogen decoding and positional information [12,28,29,30,31,32], we use the principle for a coarse-grained approach to understand phenotypic dynamics of the cells which encode environmental information. In this framework, cells combine noisy environmental inputs with prior beliefs about their internal state to update their phenotypic configuration over time. These updates subsequently act as priors for future decisions, leading to a dynamical process of learning and adaptation. Exploiting a separation of time scales between fast environmental dynamics and slower internal phenotypic dynamics at the level of probability distributions, we formulate the evolution of phenotypic states as a Bayesian updating process augmented by replication and diffusion. This yields a coarse-grained description of phenotypic evolution that does not rely on explicit molecular mechanisms but instead captures effective statistical relationships between internal and external variables. Finally, we distinguish phenotypic adaptation—the continuous dynamic process of updating internal states to minimize surprise—from cell fate, which represents the resulting stable steady-state attractors (fixed points) of this process. Differentiation is therefore interpreted as the emergence and stabilization of these attractors. To aid the reader in navigating the interdisciplinary nature of this work, we provide a comprehensive glossary (Table A1) at the end of the manuscript, which defines the core biological concepts and their precise mathematical representations.

The remainder of the paper is organized as follows. In Section 2 we introduce the Bayesian replicator-diffusion model and derive the effective Fokker-Planck description of phenotypic dynamics. Section 3 analyzes the resulting phenotypic landscapes, focusing on correlation-driven differentiation (positive or negative), sensing deterioration, and the role of proliferation in tissue homeostasis and carcinogenesis. In Section 4, we discuss the broader implications of this framework and outline directions for future work.

## 2. Materials and Methods: Theoretical Developments

### 2.1. Bayesian Inference and Cell Decision-Making

The decision of cells builds upon two important aspects, i.e., (1) internal variables (i.e., representing genes, RNA molecules, translational proteins, metabolites, receptors, and phenotypes) and the (2) external variables (i.e., ligands, chemicals, nutrients, cellular density or stress fields). Cells usually receive the information from the local microenvironment, which is later deciphered in the language of internal variables. We define the external variables as $Y\in R^{m}$ which does not evolve over time due to fast time scale and acts as an observation. The internal variables are denoted by $X\in R^{n}$. In this paper, we focus solely on the scalar case n=m=1. To formulate the adaptation process, we postulate a strict timescale separation between the environmental dynamics ($\tau_{Y}$) and the phenotypic adaptation timescale (τ). This separation allows the environment to act as a static observation during a single adaptive update. Physically, this *static microenvironment* assumption holds in two distinct limits: either the environment fluctuates extremely rapidly ($\tau_{Y}\ll \tau$), acting as a self-averaging thermodynamic bath, or it evolves extremely slowly ($\tau_{Y}\gg \tau$), acting as a quenched, frozen background. Under the timescale gap assumption, over the duration of the phenotypic update τ, the distribution of the environmental state does not meaningfully evolve. As a consequence, while the phenotypic distribution P(X,t) undergoes adaptive dynamics, the marginal distribution of the environmental variable can be treated as stationary:

(1) P(Y,t)≈P(Y),

and its low-order moments are time independent. This separation of timescales and self-averaging property justify treating P(Y) as a fixed reference distribution throughout the adaptation process. If the cell acts as a Bayesian decision maker [33], then we can write the posterior probability distribution of internal variables after an increment of time t+τ, and one can write P(X∣Y,t+τ)=P(X+δX(Y),t+τ), Table 1 shows the description of the symbol

(2) $$P\left(X+\delta X\left(Y\right),t+\tau \right)=\frac{P\left(Y|X\right)P\left(X,t\right)}{P\left(Y\right)}$$

It is crucial to note that the phenotypic displacement for an individual cell, δX(Y), depends explicitly on the specific environmental signal *Y* it just sensed. However, the density P(X,t) evolves based on the expected update. As we will show in Equation (4), the effective deterministic drift of the population emerges by averaging these individual *Y*-dependent shifts over the true microenvironmental distribution q(Y|X). Here, the likelihood, i.e., $P\left(Y|X\right)$, represents the information sampled by the cell perceived in the local microenvironment which is a perceived state or believed state sensed by the cell. Further, the conditional term has been multiplied with the ratio of the probability distribution of internal variables $P\left(X,t\right)$ perceived by the cell and the probability distribution of the believed environment state without sensing $P\left(Y\right)$. Interestingly, the likelihood $P\left(Y|X\right)$ can be associated with biological processes of microenvironmental sensing. Cells perceive their surroundings by the engagement of different biochemical processes, like polymerizing pseudopodia, translocating receptor molecules or modifying its cytoskeleton according to mechanical signals [34,35]. In this way, they estimate the aforementioned empirical likelihood. Please note that the real distribution of the environment is defined by q(Y) and the corresponding joint distribution between internal states and real environment is denoted as q(X,Y) which can be further written as

(3) $$q\left(X,Y,t\right)=P\left(X,t\right)q\left(Y|X\right)$$

To build biological intuition, it is essential to distinguish between the true and perceived microenvironmental distributions. The true distribution q(Y) describes the objective physical reality of the tissue, such as the actual absolute concentration of a circulating ligand. Conversely, the perceived distribution, encoded in the likelihood P(Y|X), represents the cell’s subjective, internal measurement of that environment. Cells construct this perception through inherently noisy and imperfect mechanisms, such as receptor–ligand binding events, intracellular signaling cascades, and cytoskeletal rearrangements. Because sensing is imperfect, a mismatch can arise between the true reality and the cell’s perception. Crucially, the phenotypic state *X* evolves based on the perceived information. If a mutation causes a receptor to fire constitutively, the cell perceives a high ligand concentration regardless of the true environmental distribution q(Y). Consequently, the cell will continuously adapt its phenotype *X* to this ‘phantom’ signal, leading to a persistent behavioral mismatch. In our framework, we formally quantify this mismatch between the true and perceived distributions as the sensing bias, which, as we show in subsequent sections, acts as a primary driver of pathological states.

Here we assume that the cell state distribution is unique and defined as $P\left(X,t\right)=q\left(X,t\right)$. At the same time, cellular adaptation with the microenvironment helps the cell to jointly cope with the cellular state and its corresponding microenvironment. Expanding the Equation (2) and by averaging upon q(Y∣X),

(4) $$\begin{array}{cc} \frac{\partial P\left(X,t\right)}{\partial t}= & \frac{1}{\tau }\int \left(\frac{P\left(Y|X\right)}{P\left(Y\right)}-1\right)P\left(X,t\right)\;q\left(Y|X\right)\;dY+\left(\mu \left(X\right)-\bar{\mu }\right)P\left(X,t\right)\\ & -v\left(X\right)\frac{\partial P\left(X,t\right)}{\partial X}+D\frac{\partial^{2}P\left(X,t\right)}{\partial {\tilde{X}}^{2}} \end{array}$$

where τ is the relaxation time which dictates how fast the phenotype density will evolve over time *t*, and δX is the phenotypic displacement due to adaptation. The $v\left(X\right)=\tau^{-1}\int \delta X\left(Y\right)q\left(Y|X\right)\;dY$ incorporates the phenotypic drift and *D* is the diffusion constant as $\sqrt{\sigma_{X}^{\prime }}$. The $\sigma_{X}^{\prime }$ is the standard deviation of the noise coming from physical diffusion which is different, as the $\sigma_{X}$ is defined as the standard deviation of the noise due to imperfect decision-making which has a different origin. Here we take into account that the decision-making or the adaptation process, replication process and diffusion act as mutually exclusive processes, and act as additive operators distinct in the hierarchy of timescales: $\tau_{\mathrm{decision-making}}<\tau_{\mathrm{diffusion}}\ll \tau_{\mathrm{replication}}$. The proliferation rate of a given phenotype is defined by μ(X), while the population’s average proliferation rate is given by $\bar{\mu }$. In Equation (4), the expression $(\mu \left(X\right)-\bar{\mu })P\left(X,t\right)$ acts as a replicator term. Biologically, this implements frequency-dependent Darwinian selection: it amplifies the density of phenotypes that proliferate faster than the average ($\mu \left(X\right)>\bar{\mu }$) and depletes those that proliferate slower. We additively combine this replicator dynamic with the Bayesian adaptation and physical diffusion processes. Moreover, for simplicity, we assume that intrinsic variable depends on the phenotypic variable in the following nonlinear quasi-steady-state way:

(5) Y=f(X)+η

where the deterministic part is described as f(X), and η is a *Gaussian* distributed noise which has mean 0 and standard deviation $\sigma^{2}$. Since the slope $f^{\prime }\left(X\right)$ reflects the underlying biophysical ‘wiring’ of the sensing network (e.g., receptor affinity or structural pathway constraints), we assume it changes on a developmental or evolutionary timescale. This timescale is orders of magnitude slower than both the rapid environmental fluctuations ($\tau_{Y}$) and the phenotypic state adaptation (τ). Consequently, throughout our dynamical analysis, ρ is treated as a fixed, quasistatic control parameter.

To derive an analytically tractable effective force from the Bayesian update term in Equation (4), the integral must be approximated. We do this by considering two distinct but mathematically complementary limits. The first approach (Case-I) explores the weak-coupling regime where the overall correlation between phenotype and environment is small (|ρ|≪1). The second approach (Case-II) relaxes the weak-correlation assumption but assumes the cell operates very close to its expected homeostatic state, capturing the local corrective dynamics. Remarkably, as shown below, both complementary approximations yield a second-order polynomial form for the effective adaptation force.

#### 2.1.1. Case-I: Weak Coupling Limit (|ρ|≪1)

There exists a time-scale separation at the distribution level, where *X* can influence *Y* while not the other way around. Moreover, we assume that the joint distribution P(X,Y) behaves like a bivariate Gaussian distribution, where we can calculate the corresponding marginals P(X) and P(Y). The latter will be both Gaussians with means $\mu_{X},\mu_{Y}$ and standard deviations $\sigma_{X},\sigma_{Y}$, respectively. The *dependence ratio* can be defined as

(6) $$r\left(X,Y\right)\;:=\;\frac{P(X,Y)}{P(X)P(Y)}\;=\;\frac{P(Y|X)}{P(Y)}.$$

Assuming that the cell is weakly coupled to the environment implies the limit |ρ|≪1 (i.e., the correlation magnitude is close to zero). Physically, this represents a regime where the environment influences the phenotypic state via subtle biases rather than imposing an immediate, deterministic locking. Because the magnitude is small, this covers both weak reinforcing (ρ≥0) and weak antagonistic (ρ≤0) feedbacks. Mathematically, this small-magnitude limit allows us to safely expand the quantity r(X,Y)−1 using the *Mehler–Hermite (Gaussian Lancaster) expansion* series expansion [36,37,38] up to the second order with respect to *X* and *Y*:

(7) $$r\left(X,Y\right)-1=\frac{P(Y|X)}{P(Y)}-1\approx \rho z_{X}z_{Y}+\frac{\rho^{2}}{2}\left(z_{X}^{2}-1\right)\left(z_{Y}^{2}-1\right)+O\left(\rho^{3}\right),$$

where the $z_{X}=\frac{X-\mu_{X}}{\sigma_{X}}$ and $z_{Y}=\frac{Y-\mu_{Y}}{\sigma_{Y}}$ are the standardized intrinsic and extrinsic random variables. $S_{X}$ is the signal-to-noise ratio of the phenotype, defined as the ratio of the mean internal state to its standard deviation: $S_{X}=\mu_{X}/\sigma_{X}$. It quantifies phenotypic fidelity how precisely a cell can maintain a specific gene expression level against noise. The quantity ${\bar{z}}_{Y}$ could be inferred from persistent deviations between the external signal statistics (true distribution of external parameter irrespective of the cell’s perception) and the internal response of a cell (i.e., cellular measurement of environmental parameter distribution, which is usually imprecise). This quantity is also the cell’s intrinsic parameter. Averaging over the real distribution of the environment q(Y∣X) in Equation (4), one can write the first term in RHS as

(8) $$\int \left(\frac{P(Y|X)}{P(Y)}-1\right)q\left(Y|X\right)\;dY=\rho z_{X}{\bar{z}}_{Y}+\frac{\rho^{2}}{2}\left(z_{X}^{2}-1\right)\left({\bar{z}}_{Y}^{2}-1\right).$$

Other terms after averaging over q(Y∣X) in Equation (4) are independent of *Y* so they vanish under the centered Gaussian average, while higher-order cumulants are neglected due to a weak-correlation limit (i.e., ρ≪1), as they have functional dependency on *X*. We define ${\bar{z}}_{Y}=\frac{\bar{Y}-\mu_{Y}}{\sigma_{Y}}$ and $\bar{Y}=\int yq\left(Y=y|X\right)\;dy$ as the true environmental average. The term ${\bar{z}}_{Y}$ represents the sensing bias term, as it quantifies the difference between true average on the environment and the cell-perceived distribution average on the environment. Now, the term in Equation (8) can be written as a second-order polynomial in *X* as

(9) $$h\left(X\right)=a+bX+cX^{2}$$

with the coefficients as

(10) $$a=\frac{\rho {\bar{z}}_{Y}\langle X\rangle }{\sigma_{X}}+\frac{\rho^{2}\left({\bar{z}}_{Y}^{2}-1\right)}{2}\left(\frac{{\langle X\rangle }^{2}}{\sigma_{X}^{2}}+1\right)=\rho {\bar{z}}_{Y}S_{X}+\frac{\rho^{2}\left({\bar{z}}_{Y}^{2}-1\right)}{2}\left(1+{\left(S_{X}\right)}^{2}\right)$$

(11) $$b=\frac{\rho {\bar{z}}_{Y}}{\sigma_{X}}-\frac{\rho^{2}\left({\bar{z}}_{Y}^{2}-1\right)\langle X\rangle }{\sigma_{X}^{2}}=\frac{\rho {\bar{z}}_{Y}}{\sigma_{X}}-\frac{\rho^{2}\left({\bar{z}}_{Y}^{2}-1\right)S_{X}}{\sigma_{X}}$$

(12) $$c=\frac{\rho^{2}\left({\bar{z}}_{Y}^{2}-1\right)}{2\sigma_{X}^{2}}.$$

We define the signal-to-noise ratio or SNR as $\frac{\langle X\rangle }{\sigma_{X}}$ and $\langle X\rangle =\mu_{X}$.

#### 2.1.2. Case-II: Operating Around the Expected Phenotypic State Limit ($z_{X}\to 0$)

In this case, we focus on cell internal states characterized around an adapted fixed point, where the corresponding fluctuations around the mean are small. This is realized by the limit of the standardized intrinsic variable $z_{X}=\left(X-\mu_{X}\right)/\sigma_{X}\to 0$. At a biological level, this is more of a homeostatic and environmentally sensitive system, where the cell reacts to external fluctuations by trying to maintain a stable internal state where the external signals do not prompt any major change in the internal state of the cell but instead trigger some corrective measures that correct the state in a subtle manner. For this kind of cellular event, one can write the dependence ratio for the $z_{X}\to 0$ limit and intrinsic-extrinsic correlation $\rho \in \left(-1,1\right)$, similar to Equation (8), as

(13) $$\begin{array}{cc} \; & r\left(X,Y\right)-1=\frac{P(Y|X)}{P(Y)}-1\approx \;r_{0}\left(z_{Y}\right)(1+\left[AS_{X}+BS_{X}^{2}\right]\\ \; & +\left[\frac{A}{\sigma_{X}}-\frac{2BS_{X}}{\sigma_{X}}\right]\;X+\left[\frac{B}{\sigma_{X}^{2}}\right]\;{\left(X\right)}^{2})\;-1+\;O\;\left({\left(\frac{X}{\sigma_{X}}-S_{X}\right)}^{3}\right) \end{array}$$

After averaging over q(Y∣X) in the Equation (13), we obtain

(14) $$\;\int \left(\frac{P(Y|X)}{P(Y)}-1\right)q\left(Y|X\right)\;dY=\tilde{r}+r_{0}\left(\left[AS_{X}+BS_{X}^{2}\right]+\left[\frac{A}{\sigma_{X}}-\frac{2BS_{X}}{\sigma_{X}}\right]\;X+\left[\frac{B}{\sigma_{X}^{2}}\right]\;{\left(X\right)}^{2}\right)\;$$

Here the constant *A* and *B* are defined by

(15) $$A=\frac{\rho }{1-\rho^{2}}{\bar{z}}_{Y}\;\&\;B=\frac{\rho^{2}}{2{\left(1-\rho^{2}\right)}^{2}}\left({\bar{z}}_{Y}^{2}-{\left(1-\rho^{2}\right)}^{2}\right)$$

where $r_{0}\left(z_{Y},\rho \right)=\frac{1}{\sqrt{1-\rho^{2}}}\exp\left(-\frac{\rho^{2}}{2(1-\rho^{2})}z_{Y}^{2}\right)\geq 0$ and $\tilde{r}=\int \left(r_{0}\left(z_{Y}\right)-1\right)q\left(Y=y|X\right)\;dy\approx r_{0}\left({\bar{z}}_{Y},\rho \right)-1$. The latter can be positive for large ρ and small ${\bar{z}}_{Y}$. From Equation (14) we can actually write a second-order polynomial similar to Case-I as

(16) $$\tilde{h}\left(X\right)=\tilde{r}+\tilde{A}+\tilde{B}X+\tilde{C}X^{2}$$

where we absorb the constants in terms of coefficients as

(17) $$\begin{array}{cc} \; & \tilde{A}=AS_{X}r_{0}+BS_{X}^{2}r_{0},\\ \; & \tilde{B}=\frac{A}{\sigma_{X}}r_{0}-\frac{2BS_{X}}{\sigma_{X}}r_{0},\\ \; & \tilde{C}=\frac{B}{\sigma_{X}^{2}}r_{0}. \end{array}$$

So, at the limit of perfect sensing (i.e., ${\bar{z}}_{Y}\approx 0$), all three coefficients in the polynomial term become

(18) $$\begin{array}{cc} \; & \tilde{A}=-\frac{\rho^{2}}{2}S_{X}^{2}r_{0}<0,\\ \; & \tilde{B}=-\frac{\rho^{2}}{\sigma_{X}}S_{X}r_{0},\\ \; & \tilde{C}=-\frac{\rho^{2}}{2\sigma_{X}^{2}}r_{0}<0. \end{array}$$

The replicator term encapsulates the population level selection acting on internal cellular states, implemented as a bias of the distribution toward phenotypes with higher proliferation or survival rates. This contribution, unlike the information acquisition represented by the Bayesian update term, reflects a Darwinian amplification of advantageous internal configurations. As the $(\mu \left(X\right)-\bar{\mu })P\left(X,t\right)$ represents differential proliferation or survival of cells as a function of their internal state *X*, one can expand this term around a fixed reference phenotype $\bar{X}$ using a Taylor series up to the second order as

(19) $$\mu \left(X\right)-\bar{\mu }=\mu^{\prime }\left(X-\bar{X}\right)+\frac{1}{2}\mu^{\prime \prime }{\left(X-\bar{X}\right)}^{2}).$$

Similarly to the Bayesian term, we expand the proliferation term up to the second-order polynomial in *X*. Now, we can combine the terms together coming from Bayesian adaptation with the proliferation term and can define a kind of effective fitness term for Case-I and Case-II. For the weak correlation function case (Case-I) and around the mean value limit (Case-II), one can rewrite the new coefficients with replication term as (for details please the S.I.)

(20) $$\begin{array}{cc} \mathrm{Case}\text{-}I & \mathrm{Case}\text{-}\mathrm{II}\\ \begin{array}{cc} h_{eff} & =\frac{1}{\tau }h\left(X\right)+\mu \left(X\right)-\bar{\mu }\\ \alpha & =\frac{b}{\tau }+\mu^{\prime }-\mu^{\prime \prime }\bar{X}\\ \beta & =\frac{a}{\tau }-\mu^{\prime }\bar{X}+\frac{1}{2}\mu^{\prime \prime }{\bar{X}}^{2}\\ \gamma & =\frac{c}{\tau }+\frac{1}{2}\mu^{\prime \prime } \end{array}\; & \begin{array}{cc} h_{eff} & =\frac{1}{\tau }\tilde{h}\left(X\right)+\mu \left(X\right)-\bar{\mu }\\ \alpha & =\frac{\tilde{B}}{\tau }+\mu^{\prime }-\mu^{\prime \prime }\bar{X}\\ \beta & =\frac{\tilde{r}}{\tau }+\frac{\tilde{A}}{\tau }-\mu^{\prime }\bar{X}+\frac{1}{2}\mu^{\prime \prime }{\bar{X}}^{2}\\ \gamma & =\frac{\tilde{C}}{\tau }+\frac{1}{2}\mu^{\prime \prime } \end{array} \end{array}$$

It is important to note a key mathematical assumption regarding these coefficients. If the cell population is undergoing macroscopic transient dynamics, the mean phenotype $\bar{X}$ evolves over time, which would render the coefficients (α,β,γ) time dependent and require solving a coupled set of integro-differential equations. However, because our primary focus is on classifying the topological stability of the phenotypic landscapes (e.g., homeostatic wells vs. explosive runaways), we employ a steady-state mean-field approximation. We treat $\bar{X}$ as the fixed, relaxed population mean. In this steady-state limit, the coefficients (α,β,γ) are strictly constant, allowing them to parameterize the effective, time-independent local landscape experienced by individual cells.

### 2.2. Effective Fokker-Planck Equation for Phenotypic Dynamics

Now, we calculate an effective force acting on the phenotype which emerges from Bayesian adaptation, proliferation and diffusion processes in the context of Fokker-Planck equation (FKE). The FKE helps us to statistically evaluate the stability, variability, and prevalence of each phenotype within a population. Robust/dominant phenotypes are identified from steady states and landscape minima, while transient probability flows report on phenotypic switching, heterogeneity, and responsiveness due to noise or environmental perturbations. Now, let us write the FKE as

(21) $$\frac{\partial P\left(X,t\right)}{\partial t}=\left(\gamma X^{2}+\alpha X+\beta \right)P\left(X,t\right)-v\left(X\right)\frac{\partial P\left(X,t\right)}{\partial X}+D\frac{\partial^{2}P\left(X\right)}{\partial X^{2}}$$

We note an important mathematical distinction regarding Equation (21). Because it incorporates an explicit replicator term $(\mu \left(X\right)-\bar{\mu })P\left(X,t\right)$, it is not a standard probability-conserving Fokker-Planck equation. Instead, P(X,t) should be interpreted as an unnormalized population density. The replicator term acts as a local reaction (source/sink) that amplifies or depletes the density based on relative fitness. For the equation to strictly conserve probability, $\bar{\mu }$ would need to be defined as the dynamically evolving instantaneous mean, $\bar{\mu }\left(t\right)=\int \mu \left(X\right)P\left(X,t\right)dX$, which introduces an integro-differential nonlinearity. However, because our analysis focuses on the stationary topological structure of the fitness landscapes, we treat $\bar{\mu }$ as a fixed reference parameter and analyze the resulting unnormalized density.

On the other hand, Equation (21) can be rewritten in a Fokker–Planck form [39] with a drift term appearing with a negative sign. We emphasize that this negative sign has already been absorbed into the coefficients of the polynomial term, i.e., the original fitness landscape has been explicitly rewritten as $-(\gamma X^{2}+\alpha X+\beta )$. With this convention, the dynamics are formulated directly in an effective energy-minimization language rather than fitness maximization. Therefore, we define an effective drift, considering conservative nature of the force, which reads

(22) $$\frac{\partial K(X)}{\partial X}=\gamma X^{2}+\alpha X+\beta ,$$

Now, the steady-state distribution of the system follows for conservative force ($\tilde{C}=0$) as

(23) $$\begin{array}{cc} \; & P_{\mathrm{ss}}\left(x\right)=\frac{1}{Z}\exp\;\left[\frac{1}{D}\left(\frac{\gamma }{12}X^{4}+\frac{\alpha }{6}X^{3}+\frac{\beta }{2}X^{2}\right)\right],\\ \; & Z=\int_{-\infty }^{\infty }e^{-U(X)/D}\;dX. \end{array}$$

So, the effective potential function is $U_{eff}$ which is defined as

(24) $$U_{eff}=-\frac{1}{D}\left(\frac{\gamma }{12}X^{4}+\frac{\alpha }{6}X^{3}+\frac{\beta }{2}X^{2}\right)$$

From the aforementioned Fokker-Planck equation, one can write an effective overdamped Langevin’s dynamics of the phenotype that describes a representative population phenotypic trajectory exploring this emergent landscape

(25) $$\frac{dX}{dt}=\frac{\gamma }{3}X^{3}+\frac{\alpha }{2}X^{2}+\beta X+\tilde{C}+\xi \left(t\right),$$

where ξ(t) is a white or Gaussian noise, where the mean of the noise is 0 while the standard deviation is defined by $\sigma_{X}^{\prime }$. Please note that Langevin equation parameters depend on the central moments of phenotypic distribution $\mu_{X}$ and $\sigma_{X}$. Out-of-equilibrium simulations would require to solve the system of Equation (25) and the corresponding moments. As stated above, in this work we are interested in the equilibrium dynamics, and the moments can be handled as parameters.

## 3. Results

### 3.1. Bayesian Adaptation Shapes Phenotypic Fitness Landscapes

We begin by analyzing the deterministic structure of the phenotypic dynamics generated by the effective fitness function derived above. In the absence of stochastic fluctuations, the overdamped Langevin equation governing the evolution of the phenotypic variable *X* reads

(26) $$\frac{dX}{dt}=K\left(X\right)=\frac{\gamma }{3}X^{3}+\frac{\alpha }{2}X^{2}+\beta X,$$

where the coefficients (α,β,γ) arise from the combined effects of Bayesian adaptation and proliferation (Equation (19)). Equation (26) defines an effective force that can be written as the gradient of an emergent potential $U_{\mathrm{eff}}\left(X\right)$,

(27) $$K\left(X\right)=-\frac{dU_{\mathrm{eff}}}{dX},\;U_{\mathrm{eff}}\left(X\right)=-\left(\frac{\gamma }{12}X^{4}+\frac{\alpha }{6}X^{3}+\frac{\beta }{2}X^{2}\right),$$

up to an additive constant. Without loss of generality, we assume the diffusion constant is D=1. The structure of $U_{\mathrm{eff}}\left(X\right)$ determines the stability, multistability, and large-amplitude behavior of phenotypic dynamics.

Fixed points and stability

The stationary phenotypic states are given by the roots of K(X)=0, which yield

(28) $$X^{*}=0,\;X_{\pm }^{*}=\frac{-3\alpha \pm \sqrt{\Delta }}{4\gamma },\;\Delta =9\alpha^{2}-48\gamma \beta .$$

The linear stability of a fixed point $X^{*}$ is determined by the sign of

(29) $$K^{\prime }\left(X^{*}\right)=\gamma {\left(X^{*}\right)}^{2}+\alpha X^{*}+\beta .$$

A fixed point is stable if $K^{\prime }\left(X^{*}\right)<0$ and unstable if $K^{\prime }\left(X^{*}\right)>0$. The sign of β controls the local stability of the origin, while γ determines whether the potential is confining at large |X|.

Classification of phenotypic landscapes

**Figure 1:**
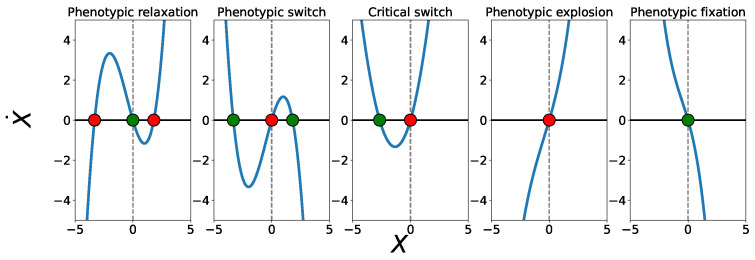
Plot of the nullclines of the phenotype at the different values of α,β and γ. Here the green color shows stable points and red color shows unstable points. The parameter values of α,β and γ follow those of phenotypic relaxation (α=1,β=−2,γ=1), phenotypic switch (α=−1,β=2,γ=−1), critical switch (α=1.5,β=2,γ=0), phenotypic explosion (α=0.5,β=2,γ=0.5) and phenotypic fixation (α=−1,β=−2,γ=−1).

Depending on the signs of (β,γ) and on the discriminant Δ, the effective potential admits a small number of qualitatively distinct configurations. We identify five canonical phenotypic landscapes, illustrated in Figure 1.

1. β<0 and γ<0. The potential has a single deep minimum at X=0 and all trajectories relax toward a stable homeostatic phenotype.
2. γ<0 and Δ>0 with β>0. Two stable minima coexist and are separated by an unstable fixed point, enabling decision-like switching between discrete phenotypic states.
3. γ≈0 or Δ≈0. One stable and one unstable fixed point coexist, corresponding to a near-critical landscape at the boundary between bistability and monostability.
4. γ>0, β<0, and Δ>0. The landscape possesses a locally stable minimum. The phenotype has a finite basin of attraction, meaning it can buffer normal fluctuations but is vulnerable to large, noise-driven perturbations that can push it over the barrier into runaway amplification.
5. β>0 and γ>0 with Δ<0. The origin is unstable, and the potential fails to confine the dynamics at large amplitudes, leading to runaway phenotypic amplification.

These regimes exhaust all possible qualitative behaviors of the cubic force in Equation (26). Importantly, they arise without assuming any specific molecular regulatory mechanisms: the landscape structure is fully determined by coarse-grained parameters encoding information flow, noise, and proliferation. Table 2 summarizes the above.

Role of the coefficients

The three coefficients (α,β,γ) admit clear mathematical and biological interpretations. The linear term β controls the local stability of the reference phenotype X=0. The quadratic term α introduces asymmetry and bias in phenotypic space, reflecting directional effects of sensing or regulation. The cubic term γ determines nonlinear saturation at large amplitude |X| and thus governs whether the landscape is confining or runaway. Subsequent sections relate these coefficients explicitly to correlation, sensing bias, phenotypic fidelity, and proliferation, and use this classification to interpret differentiation, tissue homeostasis, and carcinogenesis.

### 3.2. Correlation-Driven Emergence of Cell Fate Decisions: From Stem to Differentiated Cells

In the Gaussian setting considered here, the correlation coefficient ρ between the internal state *X* and the environmental state *Y* is directly related to the mutual information,

(30) $$I\left(X;Y\right)\;=\;-\frac{1}{2}\ln\left(1-\rho^{2}\right),$$

so that ρ can be interpreted as an information-theoretic measure of how strongly intrinsic and extrinsic variables are coupled. Large ρ corresponds to high mutual information: microenvironmental fluctuations are tightly tracked by the internal phenotype. Conversely, small ρ implies weak information flow from the environment to the phenotype and, therefore, a more decoupled and internally stabilized state.

**Figure 2:**
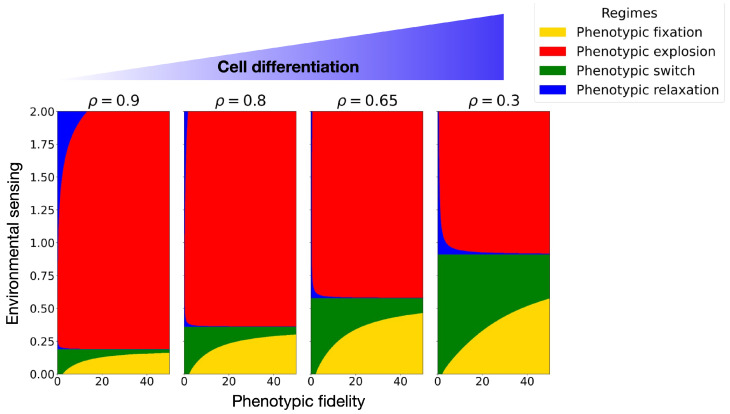
Phase space diagram of phenotypic space for positive values where we show different phases based on ${\bar{z}}_{Y}$ and the SNR ($S_{X}$). We fix the parameters $\tau =1,\sigma_{X}=1$.

To connect this information-theoretic control parameter to cell fate decisions, we construct phase diagrams in the plane spanned by the sensing bias ${\bar{z}}_{Y}$ and the phenotypic signal-to-noise ratio $S_{X}$ for different fixed values of ρ (Figure 2). Each point in this plane is classified according to the sign structure of (α,β,γ,Δ) and thus belongs to one of the four canonical phenotypic regimes defined in Table 2: phenotypic fixation (homeostatic monostable landscape), phenotypic switch (bistable decision-making landscape), phenotypic relaxation, or phenotypic explosion (runaway landscape).

For correlations close to one (ρ≈0.9), the diagram is dominated by the explosive regime (red region in Figure 2, left panel). In this limit, internal and external states are almost perfectly correlated and small changes in the microenvironment induce large phenotypic fluctuations. The effective potential becomes shallow or even unconfined, and trajectories are easily driven away from any homeostatic configuration. We interpret this regime as a coarse-grained description of highly plastic stem-like cells, whose phenotype can be strongly driven by the microenvironment.

As ρ decreases (from 0.8 to 0.65 in Figure 2), the phase diagram reorganizes. The parameter region associated with phenotypic explosion shrinks, while domains corresponding to phenotypic fixation (homeostatic monostable wells) and phenotypic switch (bistable landscapes) emerge. In these intermediate-correlation regimes, the phenotypic regulation of cells with inefficient sensing can still respond sensitively to environmental cues, but improved sensing leads to effective potentials with one or more well-defined minima. Biologically, this corresponds to partially committed progenitor states: cells begin to acquire more stable identities, yet retain a degree of plasticity through bistable decision-making between alternative phenotypic fates.

For low correlations (ρ≈0.3, rightmost panel in Figure 2), the diagram is dominated by homeostatic and bistable regimes, whereas the explosive regime shrinks significantly for high sensing bias. In this limit, the mutual information I(X;Y) is small, the effective potential wells are deep, and phenotypic fluctuations induced by the microenvironment are strongly suppressed. We associate this behavior with differentiated cells that exhibit robust fate decisions: they either maintain a single, stable phenotype (phenotypic fixation) or switch in a controlled, plastic manner between a discrete set of phenotypic states (bistable decision-making).

**Figure 3:**
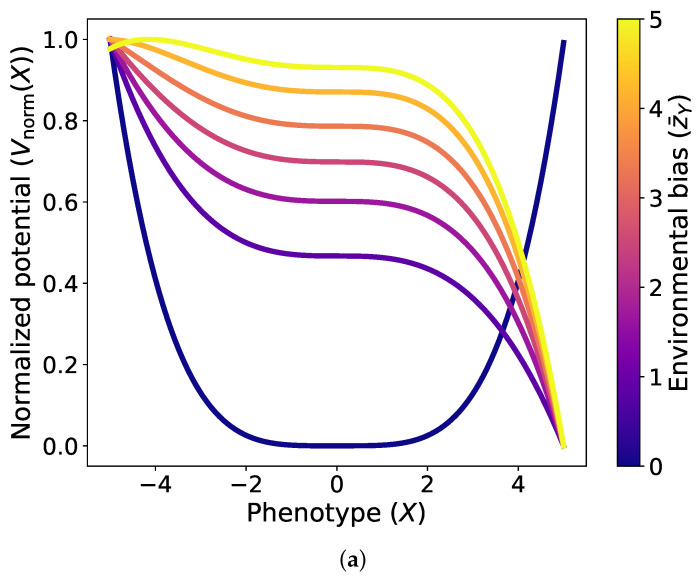

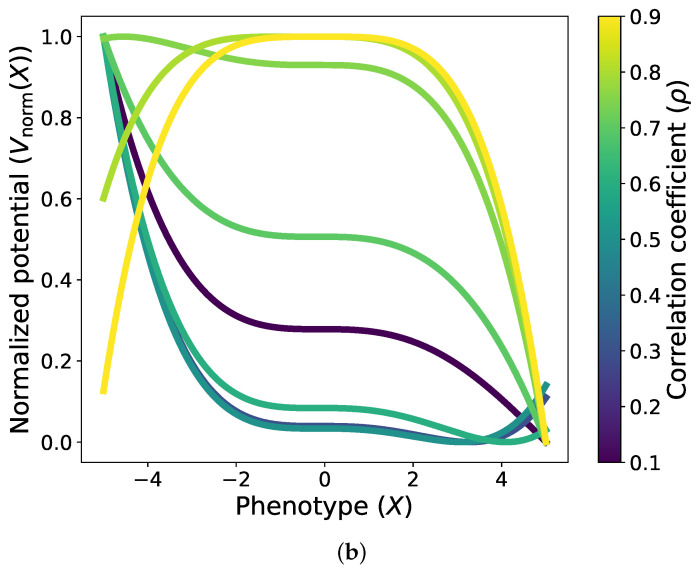
Plots of the normalized potential function of the phenotype at the limits ρ≪1 and $z_{X}\to 0$. (**a**) Plot of the normalized potential function vs. phenotype for different values of environmental bias at the limit of ρ≪1. Parameters: τ=0.01, $S_{X}=0.5$, ρ=0.0015, $\sigma_{X}=0.01$. (**b**) Plot of the normalized potential function vs. phenotype for different values of correlation coefficient at the limit of $z_{X}\to 0$. Parameters: τ=1, $S_{X}=0.25$, ${\bar{z}}_{Y}=0.5$, $\sigma_{X}=0.35$.

Another interesting point is that along the differentiation course, lowering of the correlation ρ, not only do the potential wells emerge but they also deepen. In Figure 3, we plot the potential function with respect to the phenotype in terms of $\rho ,S_{X},\sigma_{X}$ and we clearly observe the deepening of the stability regions. To generalize the result, we can analytically estimate the depth of the potential function from the conservative part of the force ($\tilde{C}=0$) as

(31) $$\begin{array}{cc} \Delta U_{\mathrm{eff}} & =U_{\mathrm{eff}}\left(X_{u}\right)-U_{\mathrm{eff}}\left(X_{s}\right)=\frac{\Delta^{3/2}}{192\;\gamma^{2}}=\frac{{\left(9\alpha^{2}-48\beta \gamma \right)}^{3/2}}{192\;\gamma^{2}}\\ \Delta U_{\mathrm{eff}} & =\frac{\sqrt{3}\;{\left(1-\rho^{2}\right)}^{5}\sigma_{X}^{4}\tau^{2}\exp\;\left(\frac{\rho^{2}{\bar{z}}_{Y}^{2}}{1-\rho^{2}}\right)}{16\rho^{4}{\left({\left(\rho^{2}-1\right)}^{2}-{\bar{z}}_{Y}^{2}\right)}^{2}}\\ \; & \;\times [-\frac{1}{{\left(1-\rho^{2}\right)}^{5}\sigma_{X}^{2}\tau^{2}}(\rho^{2}\exp\;\left(\frac{\rho^{2}{\bar{z}}_{Y}^{2}}{\rho^{2}-1}\right)[\rho^{2}S_{X}^{2}{\bar{z}}_{Y}^{4}-{\left(\rho^{2}-1\right)}^{2}{\bar{z}}_{Y}^{2}\left(2\rho^{2}S_{X}^{2}-5\right)\\ \; & \;+{\left(\rho^{2}-1\right)}^{4}\left(\rho^{2}S_{X}^{2}-8\right)-14\rho \left(\rho^{2}-1\right)S_{X}{\bar{z}}_{Y}^{3}+14\rho {\left(\rho^{2}-1\right)}^{3}S_{X}{\bar{z}}_{Y}]\\ \; & \;+8\rho^{2}{\left(1-\rho^{2}\right)}^{5/2}\exp\;\left(\frac{\rho^{2}{\bar{z}}_{Y}^{2}}{2(\rho^{2}-1)}\right)\left({\left(\rho^{2}-1\right)}^{2}-{\bar{z}}_{Y}^{2}\right)){]}^{3/2}. \end{array}$$

For large correlation ρ→1, the coupling between *X* and *Y* becomes strong and the potential barrier $\Delta U_{\mathrm{eff}}$ decreases, leading to a shallow effective potential. This facilitates frequent state switching and continuous environmental tracking without strong phenotypic preference. On the contrary, for small correlation ρ between the internal state *X* and the environment *Y*, this indicates weak coupling between sensing and phenotypic regulation. In this regime, the potential barrier $\Delta U_{\mathrm{eff}}$ is large, corresponding to a deep potential well. This reflects a stable phenotype or homeostasis associated with adaptation consolidation and strong memory. This implies that stem cells acquire new identities/phenotypes, and these phenotypes become robust in nature.

Taken together, we propose cell differentiation theory as a correlation-driven bifurcation process. High-correlation states represent stem-like cells with large microenvironmentally driven phenotypic fluctuations and explosive landscapes, whereas a progressive reduction in ρ and thus in the mutual information between *X* and *Y* leads to the emergence and stabilization of homeostatic and bistable landscapes that encode differentiated cell fates. In this view, the development and its dysregulation can be understood as deformations of a Bayesian phenotypic fitness landscape controlled by the information flow between intrinsic and extrinsic degrees of freedom.

### 3.3. Microenvironmental Sensing Deterioration as Pathway to Cancer

**Figure 4:**
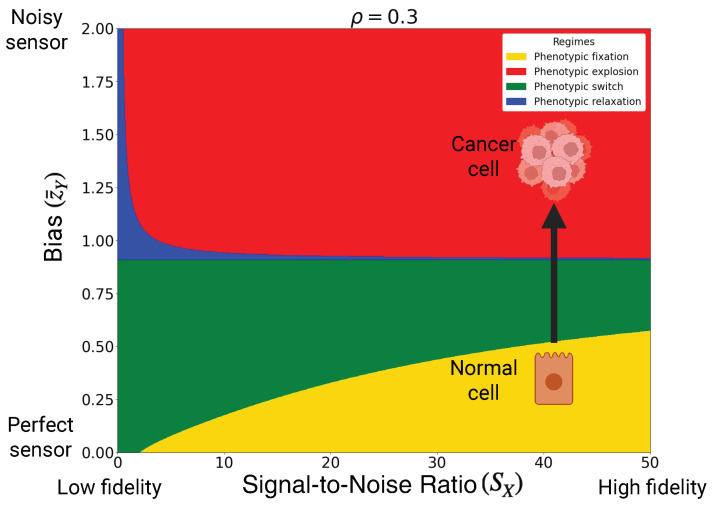
Carcinogenesis can occur when differentiated cells lose their microenvironmental sensing ability, i.e., ${\bar{z}}_{Y}$ increases, entering to the phenotypic explosion regime. The depicted sensing bias vs. SNR phase space has been calculated for correlation coefficient ρ=0.3 and for $\sigma_{X}=1$ and τ=1.

In the previous subsection, we interpreted the correlation ρ between internal and external states as an information-theoretic control parameter that drives the emergence of stem-like versus differentiated cell fates. Here, we focus on a different axis of control: the quality of microenvironmental sensing at fixed ρ as summarized in the sensing–fidelity phase diagram of Figure 4. The horizontal axis represents the phenotypic signal-to-noise ratio $S_{X}$ (phenotypic fidelity), while the vertical axis captures the sensing bias ${\bar{z}}_{Y}$, which quantifies the mismatch between the true environmental statistics and the distribution perceived by the cell. For ρ=0.3, each point in this plane again falls into one of the four canonical regimes of Table 2.

Normal, differentiated cells are expected to operate in a region of high phenotypic fidelity and accurate sensing, corresponding to large $S_{X}$ and small bias ${\bar{z}}_{Y}$ (green and yellow regions in Figure 4). In this part of the diagram, the effective fitness landscape is either homeostatic monostable (phenotypic fixation) or bistable (phenotypic switch). Both regimes are consistent with specialized cell types: they maintain a stable phenotype under perturbations, yet can undergo controlled, switch-like changes of state when appropriate signals are received. In terms of the hallmarks of cancer, such cells remain dependent on external growth cues and are sensitive to growth-suppressive signals since the shape of the landscape is still strongly constrained by the sensed microenvironment [40].

As the sensing bias ${\bar{z}}_{Y}$ increases, the perceived environment becomes progressively distorted. This deterioration of microenvironmental sensing can be interpreted as the cumulative effect of receptor loss, pathway desensitization, or persistent autocrine signaling [41]. In the phase diagram, increasing ${\bar{z}}_{Y}$ at fixed, high $S_{X}$ moves the system vertically upward. Beyond a critical threshold, the corresponding point crosses from the homeostatic or bistable region into the explosive regime, even though $S_{X}$ remains large. In this regime the effective potential loses its confining minimum and phenotypic trajectories exhibit runaway amplification: the cell no longer settles into a stable fate but instead experiences uncontrolled dynamics in phenotype space.

This transition provides a coarse-grained interpretation of several classical cancer hallmarks [42]. The loss of environmental control over the landscape may correspond to *sustained proliferative signaling* and *insensitivity to growth-suppressive cues*: once in the explosive regime, the cell’s phenotypic dynamics are dominated by internal amplification rather than by external regulation. Importantly, our framework resolves a counterintuitive paradox: how can high phenotypic fidelity coexist with phenotypic explosion? The key lies in distinguishing biological precision from accuracy. Phenotypic fidelity ($S_{X}$) governs the precision of the internal state (i.e., low internal noise in gene expression), whereas sensing bias (${\bar{z}}_{Y}$) governs its accuracy relative to the true microenvironment. Even cells with high $S_{X}$ that is, cells capable of expressing a precise, highly reproducible phenotype can become effectively autonomous if their environmental sensing becomes sufficiently biased. In this high-fidelity/high-bias regime, the cell does not suffer from random internal noise; rather, it is precisely and deterministically executing the wrong adaptive instructions, driving itself into a runaway state. In other words, cancer can be viewed as a disease of miscalibrated perception rather than merely a loss of structural phenotypic control.

### 3.4. Proliferation, Tissue Homeostasis and Carcinogenesis

So far, our analysis has focused on the phenotypic landscape generated by Bayesian adaptation alone. One might ask how cancer-like behavior can emerge before explicitly considering cell replication, which is the core clinical feature of malignancy. In our framework, the ’phenotypic explosion’ driven by sensing bias represents the critical precondition for carcinogenesis: the loss of homeostatic confinement. While clinical cancer strictly requires unchecked division, it is the prior deformation of the landscape—the removal of the restoring potential barrier—that renders the cell insensitive to growth suppressors. Once this confining boundary is lost, the cell becomes fully permissive to pathological expansion. Thus, in a growing tissue, phenotypes are subject to differential proliferation (μ(X)), which act upon this underlying informational landscape to either stabilize homeostatic states or actively amplify these runaway dynamics. In our framework, these effects are captured by the proliferation rate μ(X) entering the effective fitness polynomial

(32) $$\begin{array}{cc} \; & h_{\mathrm{eff}}\left(X\right)=\frac{1}{\tau }h\left(X\right)+\left(\mu \left(X\right)-\bar{\mu }\right)\;=\;\gamma X^{2}+\alpha X+\beta ,\;\forall \;\mathrm{case-I}\\ \; & h_{\mathrm{eff}}\left(X\right)=\frac{1}{\tau }\tilde{h}\left(X\right)+\left(\mu \left(X\right)-\bar{\mu }\right)\;=\;\gamma X^{2}+\alpha X+\beta ,\;\forall \;\mathrm{case-II} \end{array}$$

where $h_{\mathrm{eff}}\left(X\right)$ arises from Bayesian updating with proliferation process combined and $\bar{\mu }$ is the average proliferation rate. For the environmentally coupled regime (Case-II), the coefficients including proliferation (for details, please refer to the S.I.) are given by Equation (19):

(33) $$\begin{array}{ccc} \alpha =\frac{\tilde{B}}{\tau }+\mu^{\prime }-\mu^{\prime \prime }\bar{X}, & \beta =\frac{\tilde{r}+\tilde{A}}{\tau }-\mu^{\prime }\;\bar{X}+\frac{1}{2}\mu^{\prime \prime }{\bar{X}}^{2}, & \gamma =\frac{\tilde{C}}{\tau }+\frac{1}{2}\mu^{\prime \prime }, \end{array}$$

where $\mu^{\prime }=\partial_{X}\mu$ and $\mu^{\prime \prime }=\partial_{X}^{2}\mu$ are evaluated around the reference phenotype, and $\left(\tilde{A},\tilde{B},\tilde{C}\right)$ encode the Bayesian adaptation part through ρ, ${\bar{z}}_{Y}$ and $S_{X}$ (Equations (15)–(17)).

The deterministic phenotypic dynamics follow from the effective force

(34) $$\frac{dX}{dt}=K\left(X\right)=\frac{\gamma }{3}X^{3}+\frac{\alpha }{2}X^{2}+\beta X,$$

so that the homeostatic state $X^{*}=0$ is locally stable if $K^{\prime }\left(0\right)=\beta <0$ and unstable if β>0, while the sign of γ controls the large-amplitude behavior of the landscape (Table 2). In particular, the explosive, cancer-like regime is characterised by γ>0 and β>0, leading to a single unstable fixed point and a runaway trajectory in phenotype space.

Linear proliferation: the case $\mu^{\prime \prime }=0$

We first consider the simplest case of a locally linear proliferation profile around the reference phenotype,

(35) $$\mu \left(X\right)\approx \bar{\mu }+\mu^{\prime }\left(X-\bar{X}\right),\;\mu^{\prime \prime }=0.$$

In this case Equation (33) simplifies to

(36) $$\alpha =\alpha_{\mathrm{adapt}}+\mu^{\prime },\;\beta =\beta_{\mathrm{adapt}}-\mu^{\prime }\;\bar{X},\;\gamma =\gamma_{\mathrm{adapt}},$$

where we have defined the adaptation-only coefficients

(37) $$\alpha_{\mathrm{adapt}}=\frac{\tilde{B}}{\tau },\;\beta_{\mathrm{adapt}}=\frac{\tilde{r}+\tilde{A}}{\tau },\;\gamma_{\mathrm{adapt}}=\frac{\tilde{C}}{\tau }.$$

Thus, linear proliferation does not modify the large-amplitude saturation term γ (and therefore cannot by itself create or remove the explosive regime), but it *tilts* the landscape by shifting the linear and quadratic contributions.

The local stability of the homeostatic state is determined by

(38) $$K^{\prime }\left(0\right)=\beta =\beta_{\mathrm{adapt}}-\mu^{\prime }\;\bar{X}.$$

For a fixed adaptation landscape, the sign of β is controlled by the slope $\mu^{\prime }$:

- If $\beta_{\mathrm{adapt}}<0$, adaptation alone already stabilizes $X^{*}=0$. A range of values of $\mu^{\prime }>0$ preserves β<0, corresponding to robust homeostasis.
- If $\beta_{\mathrm{adapt}}>0$, adaptation alone would make $X^{*}=0$ unstable. A suitable choice of $\mu^{\prime }<0$ can further enhance instability (make β more positive).

Biologically, $\mu^{\prime }$ quantifies how proliferation responds to small deviations in phenotype. If cells deviating from the homeostatic phenotype proliferate faster than average ($\mu^{\prime }>0$ with $\bar{X}>0$), the term $-\mu^{\prime }\bar{X}$ tends to decrease β and can partially compensate for an unstable adaptation contribution. Conversely, if cells deviating from homeostasis proliferate slower ($\mu^{\prime }<0$), β is increased and the origin can be destabilized. Importantly, because γ remains equal to $\gamma_{\mathrm{adapt}}$, the presence or absence of a confining well at large |X| is still determined entirely by sensing properties (correlation ρ, bias ${\bar{z}}_{Y}$ and SNR $S_{X}$). In the linear case $\mu^{\prime \prime }=0$, proliferation therefore primarily modulates the *local* stability and bias of the landscape rather than its global confinement.

Healthy tissue homeostasis corresponds, in this language, to a regime where the adaptation part and the proliferation profile combine to yield β<0 and γ<0: perturbations of the phenotype decay and the effective potential possesses a single deep minimum (phenotypic fixation) or a controlled bistable structure (phenotypic switch). Carcinogenic transformation in the $\mu^{\prime \prime }=0$ limit requires that the adaptation parameters first drive the system into a regime with $\gamma_{\mathrm{adapt}}>0$ (loss of saturation at large |X|), and that the combined effect of adaptation and proliferation yields β>0. In that case the only fixed point at X=0 becomes repelling, and the phenotype undergoes unbounded amplification, consistent with sustained proliferative behavior that has escaped microenvironmental control.

Beyond linear proliferation: curvature $\mu^{\prime \prime }\neq 0$ and growth control

When $\mu^{\prime \prime }\neq 0$, proliferation no longer acts only as a tilt but also reshapes the large-amplitude behavior of the landscape through γ:

(39) $$\gamma =\gamma_{\mathrm{adapt}}+\frac{1}{2}\mu^{\prime \prime }.$$

A negative curvature $\mu^{\prime \prime }<0$ corresponds to saturating growth (proliferation is reduced for extreme phenotypes) and makes γ more negative, deepening the confining well and protecting against explosive dynamics. This is consistent with growth control mechanisms in healthy tissues, such as contact inhibition [43] and resource limitation [44], which penalize phenotypes that stray too far from homeostasis.

Conversely, a positive curvature $\mu^{\prime \prime }>0$ corresponds to superlinear growth (proliferation increases for extreme phenotypes) and makes γ more positive. If the adaptation part already brings the system close to the boundary between confinement and explosion, even a moderate positive $\mu^{\prime \prime }$ can flip γ to positive and push the landscape into the explosive regime. In this situation, proliferation no longer counteracts deviations from homeostasis but amplifies them, in line with the hallmarks of cancer such as sustained proliferative signaling and insensitivity to growth-suppressive cues.

In summary, proliferation shapes phenotypic landscapes at two complementary levels. With $\mu^{\prime \prime }=0$, the linear dependence of proliferation on phenotype tilts the landscape and controls the local stability of homeostatic states through β, modulating whether a given adaptive landscape supports fixation or instability. When $\mu^{\prime \prime }\neq 0$, the curvature of the proliferation profile directly alters the nonlinear saturation encoded in γ, either reinforcing tissue homeostasis (for $\mu^{\prime \prime }<0$) or facilitating carcinogenesis (for $\mu^{\prime \prime }>0$) by converting marginally confined landscapes into explosive, cancer-like ones.

### 3.5. Negative Correlation-Driven Bifurcation: The Robustness–Plasticity Trade-Off

**Figure 5:**
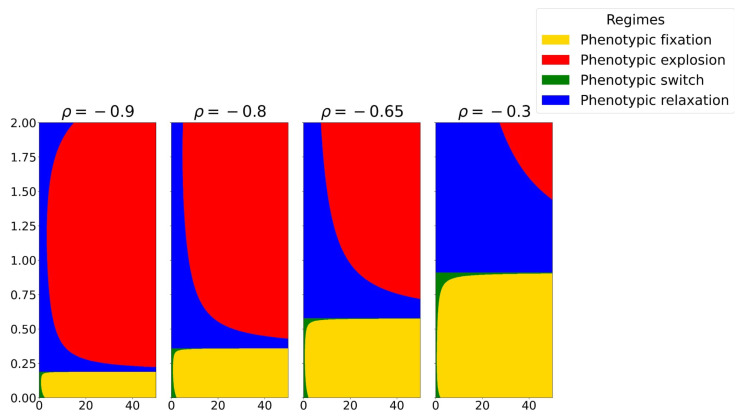
The $\left({\bar{z}}_{Y},S_{X}\right)$ plane for different negative intrinsic–extrinsic correlations. We fix the parameters $\sigma_{X}=1$ and τ=1.

So far, our analysis has focused primarily on positive correlations between intrinsic phenotypic states and extrinsic microenvironmental variables. In this regime, the phase diagrams in the $\left({\bar{z}}_{Y},S_{X}\right)$ plane display extended regions of bistability and phenotypic explosion, enabling both phenotypic plasticity and malignant runaway. Here, we investigate the complementary case of *negative* correlations, motivated by the numerical phase diagrams shown in Figure 5.

Our simulations reveal two robust qualitative effects of negative correlation. First, the region associated with phenotypic explosion (red area) is strongly reduced compared to the positive correlation case. Second, and more subtly, the bistable decision-making region (green area) is also significantly diminished. As a consequence, negative correlations promote global stability and suppress runaway dynamics but at the cost of reducing the parameter space supporting bistable phenotypic switching.

This behavior can be understood by revisiting the origin and interpretation of the correlation parameter ρ. In our model, environmental variable is described by the stochastic relation

(40) $$Y=f\left(X\right)+\eta \approx f\left(X_{0}\right)+f^{\prime }\left(X_{0}\right)\left(X-X_{0}\right)+\eta ,$$

where f(X) captures the deterministic coupling between phenotype and environment, and η is Gaussian noise. Here, the correlation coefficient between *X* and *Y* is therefore proportional to the local slope $\rho \propto f^{\prime }\left(X_{0}\right)$. Positive correlation corresponds to $f^{\prime }\left(X_{0}\right)>0$, meaning that increases in the phenotype are reinforced by environmental cues, whereas negative correlation corresponds to $f^{\prime }\left(X_{0}\right)<0$, implying an antagonistic or compensatory coupling between intrinsic and extrinsic variables.

Negative correlation thus acts as an effective negative feedback between the phenotype and environment. Our numerical analysis reveals a non-trivial, non-monotonic relationship between this antagonistic coupling and landscape stability. At intermediate negative correlations (−0.6<ρ<−0.2), the antagonistic feedback acts as a stabilizer: the cell compensates for environmental fluctuations via mechanisms such as contact inhibition, effectively deepening the potential well and suppressing runaway dynamics. However, at extreme negative correlations (ρ→−1), the system enters a regime of ‘high-gain’ instability. In this limit, the cell effectively overcompensates for even minor environmental noise, leading to violent phenotypic oscillations or runaway behavior analogous to stress-induced mutagenesis or pathological overreaction. This is reminiscent of overcompensatory systems in feedback control theory [45]. Thus, while moderate compensatory feedback promotes homeostasis, excessive antagonistic coupling drives a distinct form of phenotypic explosion.

This alternative differentiation course via negative intrinsic–extrinsic correlations cannot be the dominant regime in physiological tissues. Many well-documented cellular processes, such as epithelial-mesenchymal transition (EMT/MET) [46] or immune activation [47], rely on bistable or multistable dynamics that enable controlled phenotypic plasticity. The suppression of bistability observed for ρ<0 would therefore imply overly rigid phenotypic behavior, characterizing a subset of physiological tissues. Candidates for such tissues can be the ones that cancer occurrences are rare or nonexistent, such as heart, skeletal muscle, mature neurons and more [48].

At the same time, the stabilizing effect of negative correlation has a potentially important therapeutic implication. In the cancer-like regime, phenotypic explosion corresponds to a loss of confinement in the effective landscape, driven by biased sensing and deregulated proliferation. Introducing or enhancing negative feedback between intrinsic phenotypes and microenvironmental signals effectively pushing the system toward ρ<0 can strongly reduce the explosive region and drive the system back toward a stable, homeostatic phenotype. In this sense, negative correlation provides a theoretical mechanism for *phenotypic re-stabilization* rather than phenotypic plasticity.

## 4. Discussion

In this work, we developed a coarse-grained, information-theoretic framework in which cellular phenotypic adaptation is described as Bayesian decision-making embedded in a replicator–diffusion process. Bayesian updating of noisy environmental cues generates an effective drift term, while replication and diffusion complete an emergent Fokker–Planck description of phenotype dynamics. Specifically, these three mechanisms play distinct but mathematically complementary roles in our framework: (i) Bayesian adaptation acts as the informational drive, generating the baseline topological shape of the landscape; (ii) proliferation acts as the population-level selector, either tilting this landscape or altering its global confinement (the quartic barrier); and (iii) physical diffusion acts as the system’s ‘temperature,’ determining the rate at which cells dynamically explore this landscape and cross phenotypic barriers. The resulting effective potential defines a phenotypic fitness landscape that is not assumed *a priori* but derived from sensing fidelity, environmental bias, correlation between intrinsic and extrinsic variables, and proliferation. Within this landscape, we identified homeostatic fixation, bistable switching, and explosive regimes, and used them to interpret differentiation, tissue homeostasis, and carcinogenesis.

Within the Gaussian setting considered here, the correlation coefficient ρ between intrinsic and extrinsic states plays a central conceptual role. Because ρ directly encodes the mutual information I(X;Y), it acts as an information-theoretic control parameter for the shape of the phenotypic landscape: high correlation yields shallow or unconfined landscapes with high plasticity, whereas decreasing ρ deepens potential wells and stabilizes discrete phenotypic fates. In this view, differentiation corresponds to a correlation-driven bifurcation process in which stem-like, highly plastic cells move from an explosive regime towards robustly differentiated states as mutual information decreases. Proliferation modulates this picture at two levels: linear responses in $\mu^{\prime }\left(X\right)$ tilt the landscape and change local stability, while curvature $\mu^{\prime \prime }\left(X\right)$ alters global confinement so that superlinear growth can convert marginally stable landscapes into fully explosive ones.

This framework also provides a fresh perspective on the nature of stemness and malignancy. Classically, stem cell behavior is often viewed as an innate, hardwired epigenetic trait. However, our model reframes stemness as an emergent property of information topology: the highly plastic, ‘shallow’ landscape of a stem cell requires continuous, high-fidelity informational coupling (ρ≈1) with its microenvironment (the stem cell niche) to be maintained. If this coupling is weakened, the landscape inherently deepens into differentiated wells. Similarly, while cancer is frequently driven by single-cell mutations, it is fundamentally a disease of tissue-level organization. Our model captures this ‘tissue aspect’ not by modeling explicit spatial mechanics but by embedding tissue-level constraints into the parameters of the Fokker-Planck equation. The correlation ρ defines the cell’s integration into the tissue niche, the sensing bias ${\bar{z}}_{Y}$ quantifies the cell’s informational decoupling from its neighbors, and the proliferation curvature $\mu^{\prime \prime }$ acts as a proxy for tissue-level physical constraints like contact inhibition. Thus, carcinogenesis emerges here precisely as the breakdown of cell-tissue communication, transforming a regulated multi-cellular collective into a population of informationally autonomous, explosive agents.

Our numerical exploration of *negative* intrinsic–extrinsic correlations (ρ<0) reveals an interesting asymmetry. When ρ is negative, the region of explosive dynamics in the $\left({\bar{z}}_{Y},S_{X}\right)$ phase space becomes strongly suppressed, but the bistable decision-making region is also substantially reduced. Thus, negative correlations enhance overall robustness at the cost of strongly limiting phenotypic plasticity. This is consistent with the interpretation of ρ as the local slope of the environment-phenotype coupling in Equation (4), since ρ>0 corresponds to reinforcing feedback between phenotype and environment, whereas ρ<0 encodes an antagonistic, compensatory coupling. While such antagonistic coupling may be too rigid to account for the rich bistable phenotypic dynamics observed in physiological processes such as EMT/MET, it suggests a potential strategy for re-stabilizing pathological states.

These observations have direct implications for cancer. In our framework, carcinogenesis emerges when deteriorating microenvironmental sensing (increasing ${\bar{z}}_{Y}$) and loss of growth control (positive curvature $\mu^{\prime \prime }$) jointly drive the system into an explosive regime in which the effective potential loses its confining minimum. In this regime, cells become effectively autonomous from growth-regulating cues: their phenotypic trajectories are dominated by internal amplification rather than external regulation, consistent with hallmarks such as sustained proliferative signaling and insensitivity to growth-suppressive cues [40]. Crucially, this malignant-like behavior does not require low phenotypic fidelity: even cells with high signal-to-noise ratio $S_{X}$ and therefore precise, reproducible phenotypes—can undergo phenotypic explosion if their environmental sensing becomes sufficiently biased. Therapeutically, our results suggest two complementary routes to restore control: (i) reducing effective sensing bias or introducing antagonistic feedback to shift the effective ρ towards less explosive regimes, and (ii) enforcing saturating or sublinear proliferation (negative $\mu^{\prime \prime }$) to deepen confining wells and penalize extreme phenotypes. The former bears similarities to the ideas of differentiation therapy [49] and feedback-restoring therapies [50]. Both approaches aim to push the system back from the explosive region of the phase diagram into homeostatic or controlled bistable regimes.

The framework also provides a natural lens on exploration–exploitation trade-offs in cell fate decision-making [7]. High-correlation, weakly confining landscapes support large phenotypic excursions and multiple accessible fates: stem-like or progenitor cells in this regime “explore” phenotype space, using microenvironmental cues to sample a broad range of states. As correlation decreases and the landscape develops deeper wells, cells progressively “exploit” a reduced subset of phenotypes, committing to robust differentiated fates. Bistable landscapes occupy an intermediate regime, balancing exploration (via occasional switching) with exploitation (via stable wells) and thereby capturing controlled phenotypic plasticity. This interpretation is consistent with observations that multipotent lineages exhibit stronger drift and diffusion in high-dimensional state space than fully committed cells, and that state-dependent stochasticity is essential for maintaining non-equilibrium developmental dynamics.

Our framework offers distinct statistical signatures of the transition from homeostasis to malignancy. Increasing sensing bias (${\bar{z}}_{Y}$) flattens the homeostatic potential well. Experimentally, this appears as an increase in the variance (width) of the gene or transcriptional expression distribution in single-cell snapshot data. Interestingly, several works support the observation that cancer cell gene or RNA expressions broaden their distributions [51,52] or the entropy of the corresponding signaling networks increases [53]. Potentially, reasons for this distribution broadening in cancer are paired with a loss of mutual information between the environment and the phenotype as suggested by [54]. In the extreme case of constitutive activation (e.g., KRAS/EGFR mutations), signaling output becomes partially decoupled from ligand concentration. Finally, the onset of the effective potential flattening can be detected as ‘critical slowing down’an increase in the autocorrelation time of single-cell fluctuations prior to the malignant transition.

Beyond biological interpretation, our results suggest concrete ways to inform learning algorithms for cell dynamics, particularly neural stochastic differential equation frameworks such as scDiffEq [55]. In those models, drift and diffusion are represented by neural networks and trained from single-cell data using optimal-transport losses. Our theory provides a family of analytically tractable drift fields cubic forces derived from Bayesian adaptation and proliferation that can serve as inductive biases or regularization targets. Conversely, neural SDE models can be used to infer empirical drift and diffusion fields from high-dimensional data and then project them onto low-dimensional phenotypic coordinates, allowing the direct estimation of effective coefficients (α,β,γ) and of the inferred $\rho ,{\bar{z}}_{Y},S_{X},\mu \left(X\right)$ from data. Specifically, applying these tools to cross-sectional snapshot data should reveal a topological inversion of the inferred drift field: from a confining cubic function in healthy tissues (γ<0) to a convex, runaway profile in cancerous states (γ>0). In this way, our analytic framework and data-driven neural differential equation approaches become mutually constraining: theory guides model architecture and regularization, while learned dynamics provide empirical tests of the predicted landscape regimes. A similar endeavor, inspired by the ideas of adaptation and learning, is the development of geodesic learning [56].

Several limitations of the present framework point to important directions for future work. First, we assumed Gaussian statistics between intrinsic and extrinsic variables, which allowed us to identify correlation with mutual information in closed form and to derive a simple cubic force. Non-Gaussian or multimodal statistics would generate more rugged, hierarchical landscapes and may be essential to capture complex fate trees and oscillatory dynamics. Second, we restricted ourselves to one-dimensional phenotypic and environmental variables; real single-cell state spaces are high dimensional, and extending the theory to higher dimensions will be necessary to connect more directly to transcriptomic or epigenetic data and to exploit the full power of neural SDE learning frameworks. Third, we neglected explicit cell–cell interactions and spatial mechanics, which are known to feed back on phenotypes and may qualitatively alter the landscape at the tissue level. Additionally, we assumed a constant diffusion coefficient *D*. In highly heterogeneous tissues, physical noise is often state dependent (D(X)), which would introduce noise-induced drift terms that actively deform the landscape a phenomenon that warrants dedicated exploration. Finally, our therapeutic and algorithmic implications are conceptual: translating effective parameters such as ρ, ${\bar{z}}_{Y}$ or $\mu^{\prime \prime }$ into concrete molecular interventions or into practical regularization schemes for learning algorithms will require careful calibration against experimental data. Despite these limitations, the present work offers a compact, interpretable scaffold on which more detailed mechanistic models and data-driven neural differential equation approaches can be systematically built.

## Figures and Tables

**Table 1:** Table of symbols.

Symbol	Description
*X*	Internal/phenotypic random variable
*Y*	External/environmental random variable
*x*	Internal/phenotypic states realization
*y*	External/environmental states realization
P(X∣Y)	Posterior probability distribution of *X* given *Y*
P(Y∣X)	Likelihood of perceived environmental state given internal state
P(X,t)	perceived probability distribution of *X* at time *t*
P(Y)	Marginal perceived probability distribution of *Y*
q(Y)	True distribution of microenvironment *Y*
q(X,Y)	True joint distribution between *X* and *Y*
q(Y∣X)	True conditional distribution of *Y* given *X*
τ	Relaxation time for adaptation
δX(Y)	Phenotypic displacement due to Bayesian learning.
v(X)	Drift velocity of the phenotype during adaptation.
*D*	Diffusion constant ($\sqrt{\sigma_{X}^{\prime }}$) modeling physical diffusion noise
$\sigma_{X}$	Standard deviation of noise from imperfect decision-making
μ(X)	Proliferation (or growth) rate
$\bar{\mu }$	Average proliferation rate $\bar{\mu }=\int \mu \left(X\right)P_{ss}\left(X\right)dX$
f(X)	Deterministic term of environmental relation Y=f(X)+η
η	Gaussian distributed microenvironmental noise
ρ	Intrinsic–extrinsic (X,Y) correlation coefficient
$\bar{Y}$	Expected *Y* over true distribution q(Y)
$\mu_{Y}$	Expected *Y* over perceived distribution p(Y)
$\mu_{X}$	Expected *X* over the distribution of *X*
$z_{X}=\left(X-\mu_{X}\right)/\sigma_{X}$	Standardized internal variable
$z_{Y}=\left(Y-\mu_{Y}\right)/\sigma_{Y}$	Standardized external variable
${\bar{z}}_{Y}$	Expected $z_{Y}$ under the true conditional q(Y∣X)
$S_{X}=\langle X\rangle /\sigma_{X}=\mu_{X}/\sigma_{X}$	signal-to-noise ratio (SNR) for internal variable
$P_{\mathrm{ss}}\left(X\right)$	Steady-state probability distribution of phenotypes
*Z*	Normalization constant (partition function)

**Table 2:** Parameter constellations that define phenotypic regimes and the corresponding potential landscapes.

Phenotypic Regime	Δ	β	α	γ	Potential Landscape
Phenotypic relaxation	>0	<0	any	>0	Two barriers, finite well
Phenotypic switch	>0	>0	any	<0	Two wells, finite barrier
Critical switch	=0	any	any	→$0^{+}$	Vanishing barrier
Phenotypic explosion	<0	>0	>0	>0	Runaway, no minimum
Phenotypic fixation	<0	<0	<0	<0	Single deep global well

## Data Availability

All computational analyses and modeling code supporting this study are available upon request.

## Appendix A

### Appendix A.1. Derivation of the Effective Fitness Term $h_{\mathrm{eff}}\left(X\right)$ and Its Coefficients

The full contribution to the phenotypic dynamics of the cell is the sum of two terms as follows:

1. The **Bayesian adaptation term** (information-acquisition/sensing), which after the *Mehler–Hermite* expansion and averaging over the true environmental measure q(Y∣X) becomes a quadratic polynomial in the internal state *X* which can approximated as
  - Case-I (ρ≪1): $\frac{1}{\tau }h\left(X\right)$ with $h\left(X\right)=a+bX+cX^{2}$
  - Case-II ($z_{X}\to 0$): $\frac{1}{\tau }\tilde{h}\left(X\right)$ with $\tilde{h}\left(X\right)=\tilde{r}+\tilde{A}+\tilde{B}X+\tilde{C}X^{2}$
2. The **replicator (proliferation) term** $\mu \left(X\right)-\bar{\mu }$, which is expanded to second order around the current population mean $\bar{X}$ and can be approximated as
  (A1) $$\mu \left(X\right)-\bar{\mu }=\mu^{\prime }\left(X-\bar{X}\right)+\frac{1}{2}\mu^{\prime \prime }{\left(X-\bar{X}\right)}^{2}+O\left({\left(X-\bar{X}\right)}^{3}\right),$$
  where $\mu^{\prime }=\frac{d\mu }{dX}{|}_{\bar{X}}$, $\mu^{\prime \prime }=\frac{d^{2}\mu }{dX^{2}}{|}_{\bar{X}}$, and $\bar{\mu }=\mu \left(\bar{X}\right)$.

We now collect powers of *X* after substituting the two expansions until the second-order term approximation.

### Appendix A.2. Case-I (p≪1)

(A2)
$$\begin{array}{cc} h_{\mathrm{eff}}\left(X\right) & =\frac{1}{\tau }\left(a+bX+cX^{2}\right)+\mu^{\prime }\left(X-\bar{X}\right)+\frac{1}{2}\mu^{\prime \prime }\left(X^{2}-2\bar{X}X+{\bar{X}}^{2}\right)\\ \; & =\left(\frac{c}{\tau }+\frac{1}{2}\mu^{\prime \prime }\right)X^{2}+\left(\frac{b}{\tau }+\mu^{\prime }-\mu^{\prime \prime }\bar{X}\right)X+\left(\frac{a}{\tau }-\mu^{\prime }\bar{X}+\frac{1}{2}\mu^{\prime \prime }{\bar{X}}^{2}\right). \end{array}$$

Hence the coefficients are

(A3) $$\begin{array}{cc} \alpha & =\frac{b}{\tau }+\mu^{\prime }-\mu^{\prime \prime }\bar{X},\\ \beta & =\frac{a}{\tau }-\mu^{\prime }\bar{X}+\frac{1}{2}\mu^{\prime \prime }{\bar{X}}^{2},\\ \gamma & =\frac{c}{\tau }+\frac{1}{2}\mu^{\prime \prime }. \end{array}$$

Case-II (z_X_ → 0):

(A4) $$\begin{array}{cc} h_{\mathrm{eff}}\left(X\right) & =\frac{1}{\tau }\left(\tilde{r}+\tilde{A}+\tilde{B}X+\tilde{C}X^{2}\right)+\mu^{\prime }\left(X-\bar{X}\right)+\frac{1}{2}\mu^{\prime \prime }\left(X^{2}-2\bar{X}X+{\bar{X}}^{2}\right)\\ \; & =\left(\frac{\tilde{C}}{\tau }+\frac{1}{2}\mu^{\prime \prime }\right)X^{2}+\left(\frac{\tilde{B}}{\tau }+\mu^{\prime }-\mu^{\prime \prime }\bar{X}\right)X+\left(\frac{\tilde{r}}{\tau }+\frac{\tilde{A}}{\tau }-\mu^{\prime }\bar{X}+\frac{1}{2}\mu^{\prime \prime }{\bar{X}}^{2}\right). \end{array}$$

Hence

(A5) $$\begin{array}{cc} \alpha & =\frac{\tilde{B}}{\tau }+\mu^{\prime }-\mu^{\prime \prime }\bar{X},\\ \beta & =\frac{\tilde{r}}{\tau }+\frac{\tilde{A}}{\tau }-\mu^{\prime }\bar{X}+\frac{1}{2}\mu^{\prime \prime }{\bar{X}}^{2},\\ \gamma & =\frac{\tilde{C}}{\tau }+\frac{1}{2}\mu^{\prime \prime }. \end{array}$$

The effective fitness is therefore

(A6) $$h_{\mathrm{eff}}\left(X\right)\;=\;\left\{\begin{array}{cc} \frac{1}{\tau }h\left(X\right)+\mu \left(X\right)-\bar{\mu } & (\mathrm{Case}\;I)\\ \frac{1}{\tau }\tilde{h}\left(X\right)+\mu \left(X\right)-\bar{\mu } & (\mathrm{Case}\;\mathrm{II}) \end{array}\right.$$

## Appendix B. Glossary

**Table A1:** Glossary of terms and symbols used in the theoretical framework.

Symbol/Term	Definition	Biological Interpretation
**Phenotypic Adaptation**	The dynamic process of updating the internal state *X* over time (defined by the velocity field dX/dt).	The continuous adjustment of gene expression or metabolic states in response to environmental inputs to minimize "surprise" (prediction error).
**Cell Fate**	A stable fixed point $X^{*}$ of the phenotypic dynamics where dX/dt=0 and $K^{\prime }\left(X^{*}\right)<0$.	A robust, differentiated identity (e.g., a specific cell type) that remains stable against small fluctuations.
**Phenotypic Fidelity** ($S_{X}$)	The signal-to-noise ratio of the internal state, defined as $S_{X}=\langle X\rangle /\sigma_{X}$.	Represents the **precision** of the phenotype. High fidelity implies low internal noise in gene expression or signaling relative to the mean level.
**Sensing Bias** (${\bar{z}}_{Y}$)	The standardized mismatch between the true environmental mean and the cell’s perceived mean.	Represents accuracy or **sensing fidelity** (inversely). High bias indicates poor sensing fidelity, where the cell is “miscalibrated” and perceives a microenvironment that differs from reality (e.g., due to receptor saturation).
**Correlation** (ρ)	The correlation coefficient between the internal phenotype *X* and the external signal *Y*.	Quantifies the **information coupling** between the cell and its niche. ρ≈1 implies perfect tracking (stem-like plasticity), while ρ≪1 implies decoupling (differentiation).
**Phenotypic Fixation**	A landscape regime where the effective potential $U_{eff}\left(X\right)$ has a single deep minimum (γ<0).	**Phenotypic Homeostasis.** The cell is locked into a single unconditionally stable phenotype, who strongly resists to any perturbations.
**Phenotypic Switch**	A landscape regime with two stable minima separated by a barrier (bistability).	**Plasticity/Decision-Making.** The cell can toggle between discrete states (e.g., epithelial–mesenchymal transition) driven by noise or signals.
**Phenotypic Relaxation**	A monostable landscape regime (γ>0, β<0, Δ>0) characterized by a local potential well with a finite basin of attraction.	**Conditional Phenotypic Confinement.** A differentiated cell state that is stable under normal noise microenvironment. Severe noise spikes or environmental stress can push the cell over the barrier, leading to a loss of phenotypic confinement.
**Critical Switch**	A landscape regime with marginal stability (γ≈0 or Δ≈0).	**Tipping Point.** The boundary between a stable differentiated state and bistability, often observed during rapid developmental transitions.
**Phenotypic Explosion**	A landscape regime where the potential loses confinement (γ>0) and allows runaway trajectories.	**Malignancy/Carcinogenesis.** The loss of homeostatic control where the phenotype drifts unboundedly, often driven by high sensing bias or superlinear proliferation.
**Replicator Term**	The growth rate term $(\mu \left(X\right)-\bar{\mu })P\left(X,t\right)$ in the Fokker-Planck equation.	**Darwinian Selection.** The amplification of specific phenotypes based on their reproductive fitness, distinct from the Bayesian “learning” drift.
**Proliferation Curvature** ($\mu^{\prime \prime }$)	The second derivative of the proliferation rate evaluated around the population mean.	**Tissue-Level Constraints. **$\mu^{\prime \prime }<0$ implies resource limitation or contact inhibition (stabilizing), while $\mu^{\prime \prime }>0$ implies superlinear, runaway growth (destabilizing).
